# Pulmonary Impedance and Wave Reflections in Adults with Mitral Stenosis: Immediate and Follow-Up Effects of Balloon Valvuloplasty

**DOI:** 10.1007/s10439-024-03661-3

**Published:** 2024-12-15

**Authors:** Chih-Tai Ting, Jaw-Wen Chen, Mau-Song Chang, Frank C.-P. Yin

**Affiliations:** 1 Cardiovascular Center, Taichung Veterans General Hospital, Taichung, Taiwan; 2 Department of Medical Research, Veterans General Hospital, Taipei, Taiwan; 3 Department of Medicine and Cardiovascular Research Center, National Yang Ming University School of Medicine, Taipei, Taiwan; 4 Cardiology Division, Department of Medicine, Veterans General Hospital, Taipei, Taiwan; 5 Present Address: Department of Biomedical Engineering, Washington University, St. Louis, MO USA; 6 Present Address: Division Cardiovascular Medicine, Department of Internal Medicine, Department of Medical Research and Cardiovascular Research Center, Taipei Medical University Hospital, Taipei, Taiwan; 7 Present Address: Reshining Clinic, Taipei, Taiwan

**Keywords:** Pulmonary hypertension, Pulmonary impedance, Wave reflections, Mitral stenosis, Balloon valvuloplasty

## Abstract

**Purpose:**

We compared adults with mitral stenosis (MS) to 8 controls (CONT) to see how pulmonary impedance and wave reflections differ at baseline and after balloon valvuloplasty.

**Methods:**

We separated the MS patients into groups according to mean pulmonary artery pressure: moderate (MOD; ≤ 26 mmHg, n = 21) and high (HIGH; > 26 mmHg, n = 33). We made baseline high-fidelity measurements in all patients, in the MS groups after vasodilation with nitroprusside, immediately and 4 months after balloon valvuloplasty.

**Results:**

Comparing MOD vs CONT, using the Kruskal-Wallis test with Bonferroni correction, reveals evidence for higher baseline input resistance (R) (489 vs 205 dyne-sec/cm^5^, P = 0.07); first harmonic of impedance modulus (Z_1_) (97.3 vs 27.6 dyne-sec/cm^5^, P = 0.01); first zero crossing of impedance phase angle (F_0_) (4.49° vs 2.19°, P = 0.02) but no difference in wave reflection index (P_b_/P_f_). Baseline HIGH vs CONT comparisons reveal stronger evidence and larger differences than MOD for R (995 vs 205, P < 0.001); Z_1_ (151 vs 27.6, P < 0.001); F_0_ (5.25 vs 2.19, P < 0.001); as well as P_b_/P_f_ (0.69 vs 0.42, P < 0.001). Responses to nitroprusside and valvuloplasty are also greater in the HIGH than MOD, but the HIGH parameters still differ from the CONT. Four months after valvuloplasty there is evidence for reverse remodeling in both groups. Further analyses reveal that sinus rhythm and younger age are potentially important factors for remodeling.

**Conclusion:**

MS causes alterations in pulmonary hemodynamics that differ according to pressure levels. These changes are only partially reversed immediately after valvuloplasty. There is evidence for reverse remodeling 4 months afterwards.

## Introduction

Mitral stenosis (MS) is a common form of valvular heart disease representing about 12% of all the single-valve cardiac diseases [1]. It most commonly is the sequela of rheumatic fever but there are congenital as well as other etiologies [1]. The prevalence of MS is low in developed countries. It is estimated to be 0.1% in the U.S. but is more than an order of magnitude higher in Asia and Africa [1–6]. There is a significantly higher prevalence in women (1.6%) than in men (0.4%) [7].

The valvular manifestations of rheumatic fever are slowly progressive and do not become apparent for decades or more after the initial disease. Over years the leaflets thicken and become less pliable, chordae and subvalvular structures become thicker and the commissures may fuse thereby decreasing the size of the mitral orifice [5, 8]. This resulting partial obstruction causes enlargement and increased pressure in the left atrium. This is transmitted proximally and eventually results in pulmonary hypertension with its deleterious effects on the right ventricle and other organ systems. Once severe symptoms occur and/or the valve becomes moderately to severely stenotic at the well-accepted threshold valve area of < 1.5 cm^2^, it is necessary to intervene to relieve the stenosis [2, 5]. This can be done invasively by closed or open valvuloplasty, noninvasively by percutaneous balloon valvuloplasty or, as a last resort, valve replacement**.**

Because of the deleterious effects of MS on the pulmonary vascular system, it is important to have a comprehensive understanding of the system. Most of the existing literature is limited to the standard steady-state hemodynamic measurements of pulmonary pressures, flow and resistance [9–20]. More comprehensive insight about the pulmonary vasculature can be obtained by measuring pulsatile hemodynamics, e.g. impedance and wave reflections [21–29]. The additional information provided includes the following: (1) the low frequency portion of the impedance modulus is a measure of most of the pulsatile load on the right ventricle; (2) the highest frequency portion of the impedance modulus, characteristic impedance, quantifies the mechanical properties of the most proximal region of the vasculature; (3) the first zero-crossing of the impedance phase angle is a manifestation of the main site of wave reflections and the wavespeed; (4) the magnitude and timing of the reflected pressure wave are key determinants of the measured pressure; (5) the ratio of oscillatory to total power is a manifestation of the efficiency with which the pulsatile energy of the ventricle is transferred to forward flow.

There are, to our knowledge, only 4 studies of pulmonary impedance in patients with MS [30–33]. Each of them has a severe limitation precluding definitive interpretation. Three of them had too few patients to allow robust statistical analysis. The fourth had an adequate number and included data after balloon valvuloplasty, but did not have a normal group for comparison [31]. There are no reports on follow-up after valvuloplasty.

This study examines pulmonary impedance and wave reflections in a large number of patients with MS who have a wide range of pulmonary pressures. They are studied before, immediately following, and at follow-up after balloon valvuloplasty. The main purposes of this study are to test the following hypotheses: (1) compared to a control group the baseline hemodynamic alterations are different in those grouped by moderately elevated as opposed to highly elevated pressures; (2) valvuloplasty has a different effect on the groups and does not immediately reverse the abnormalities in either group; (3) after valvuloplasty reverse remodeling occurs over time in some patients.

## Materials and Methods

### Patient Selection

All patients selected for this study were ethnic Chinese residing in Taiwan. A control group, henceforth designated CONT, was comprised of 8 adults undergoing diagnostic right heart catheterization for electrophysiological testing. None had demonstrable hemodynamic abnormalities or evidence of congenital or valvular heart disease. The other study patients were adults with MS, as demonstrated on outpatient two-dimensional echocardiographic examinations, who were referred for cardiac catheterization. Patients were excluded from the study according to the following criteria:

- More than moderate valvular regurgitation.
- Concomitant aortic or tricuspid stenosis.
- Left atrial thrombus.
- Idiopathic pulmonary hypertension.
- Chronic bronchial asthma or chronic obstructive pulmonary disease.
- Significant coronary artery disease.
- Medications which influence pulmonary pressure and pulmonary function such as bronchodilators, beta-blockers, theophylline, or pulmonary antihypertensive drugs.

We grouped the MS patients according to mean pulmonary pressure (MPAP). Rather than using arbitrary cutoffs to delineate the ranges, we used the prognostic thresholds found by unbiased classification and regression tree analysis in a large cohort of patients with a range of pulmonary artery pressures [34]. That study found that MPAP in the range 17 to 26 mmHg was an independent predictor of poor survival. Accordingly, we divided the MS patients into moderately (MOD) or highly (HIGH) elevated pressure groups based on 17 < MPAP ≤ 26 mmHg or MPAP > 26 mmHg, respectively.

### Equipment

#### Echocardiography

For all the patients with MS, we performed two-dimensional echocardiograms using a Hewlett-Packard Model 77020A ultrasound system (Hewlett-Packard, Inc., Andover, MA) to assess the cardiac structures and to determine the presence of left atrial thrombus. We then switched to the color-Doppler mode to qualitatively assess the presence and severity of mitral regurgitation and the continuous-wave Doppler mode to estimate mitral valve area (MVA).

#### High-Fidelity Hemodynamics

To obtain-high fidelity hemodynamic data we used a catheter incorporating two micromanometers and an electromagnetic flow velocity sensor (VPC-684D, Millar Instruments Co., Houston, TX). The velocity sensor was connected to a flowmeter (model BL-613, Biotronex Laboratories, Silver Spring, MD). We positioned the catheter with the distal pressure and flow sensors in the proximal main pulmonary artery and the proximal pressure sensor in the right ventricle.

### Study Conditions

#### Baseline (BAS)

To avoid any contrast agent induced effects, we made all hemodynamic measurements prior to injection of contrast agents. We first measured pressures using a fluid-filled catheter and collected blood samples from appropriate sites for oximetry. Then we replaced the diagnostic catheter with the Millar catheter. We recorded the high-fidelity analog pressure and flow velocity signals on tape (Hewlett Packard 3968-A) for off-line analysis.

#### Nitroprusside (NP)

In the patients with MS, we first obtained baseline high-fidelity hemodynamic data. To assess the role of nonspecific vasoconstriction, we then infused sodium nitroprusside beginning at a dose of 0.25 mcg/kg/min. After 3 min at each infusion level, we measured pulmonary artery pressure. Then we doubled the dose and made measurements again after 3 min. We increased the infusion until the pressure either fell into the normal range or until an infusion of 2 mcg/kg/min was achieved. At that time we acquired hemodynamic data.

#### Percutaneous Mitral Balloon Valvuloplasty (PMBV)

Since all the patients had moderate to severe MS [2, 5], we performed PMBV with an Inoue balloon employing standard percutaneous techniques [16, 35]. To avoid patient fatigue, this procedure was done shortly after (mean 5.24, range 0–16, SD 4.4 days) obtaining the baseline data. Immediately before and after PMBV, we collected blood samples for oximetry. We assessed the extent of atrial septal defect (ASD) by the ratio of pulmonary to systemic blood flow (Q_p_/Q_s_) based on the oximetry data using the Fick equation. Shortly after PMBV (usually within a day), we repeated an echocardiogram to obtain estimates of MVA and ASD. The PMBV was deemed successful if the following inclusion criteria were met: (a) MVA increased to > 1.5 cm^2^ or ≥ 50% of its preprocedure value [15, 36]; (b) there was no or trivial ASD, i.e. Q_p_/Q_s_ < 1.1 (confirmed visually by echocardiography); and (c) there was less than moderate mitral regurgitation (MR) as visualized by echocardiography. In those with successful outcomes, i.e. the PMBV cohort, we obtained high-fidelity hemodynamic measurements as described above.

#### Post-percutaneous Mitral Balloon Valvuloplasty (PPMBV)

To assess short-term follow-up effects in the pulmonary vasculature we restudied some patients a few months (range 2.6–7.0, mean 4.10, SD 1.23 months) after they underwent PMBV. We first performed a diagnostic catheterization with oximetry to assess the extent of any ASD. We then obtained high-fidelity hemodynamic data as before. For these patients MVA and ASD were also assessed by echocardiography performed either the day prior to or the day after the hemodynamic study. To focus on the vascular effects, we included only patients who did not have restenosis as evidenced by either an MVA < 1.5 cm^2^ or a > 50% loss of the initial gain in MVA, [15, 36–38]. We used the same inclusion criteria for ASD and MR as for the PMBV condition.

### Study Cohorts

Applying the exclusion criteria resulted in 59 MS patients enrolled in the study. Poor quality of the flow signal (as explained in the “Calculations and Data Analysis” section below) in 3 and inability to properly position the sensors resulted in baseline (BAS) cohorts of 21 in the MOD and 33 in the HIGH. The cohorts in the NP condition (19 in MOD and 25 in HIGH) were smaller due to poor signal quality in seven, too low a pressure in two and one refusal. Of the BAS cohorts, we enrolled 14 in the MOD and 26 in the HIGH for PMBV. Among these PMBV was not done for a variety of reasons, including the presence of left atrial thrombus, inability to cross the mitral valve and patient refusal. Of those who underwent PMBV, many were excluded from the present study. The vast majority were excluded because they either did not meet the inclusion criteria for ASD and MR (described in the “Study Conditions” section above) or had poor quality signals. A few were excluded for other reasons. The resultant cohorts for the PMBV condition numbered 10 in the MOD and 15 in the HIGH. For similar reasons, the resultant cohorts for the PPMBV condition numbered 8 in the MOD and 14 in the HIGH. Some anthropometric and clinical data for the various cohorts are listed in Table 1.

**Table 1:** Anthropometric and heart rhythm data (upper portion) for the control group (CONT) and for the cohorts in the various study conditions of the moderately (MOD) and highly (HIGH) elevated pulmonary pressure mitral stenosis groups. P values for the differences between groups for the pertinent statistical analysis (lower portion)

	BAS			NP		PMBV		PPMBV	
	CONT (n = 8)	MOD (n = 21)	HIGH (n = 33)	MOD (n = 19)	HIGH (n = 25)	MOD (n = 10)	HIGH (n = 15)	MOD (n = 8)	HIGH (n = 14)
Age (years)	37.0 32.3 ~ 38.0	55.0 45.5 ~ 59.0	44.0 36.0 ~ 53.5	52.0 45.0 ~ 59.0	43.0 36.0 ~ 52.5	56.0 45.8 ~ 67.8	43.0 37.0 ~ 52.0	58.0 47.5 ~ 68.5	42.5 34.8 ~ 53.0
M\F	7\1	11\10	9\24	10\9	5\20	6\4	1\14	5\3	3\11
SR\AF	8\0	5\16	16\17	5\14	12\13	2\8	9\6	2\6	6\8
Height (cm)	167.5 160.0 ~ 172.0	161.0 154.3 ~ 166.3	158.5 153.8 ~ 166.0	161.0 153.5 ~ 166.0	158.0 153.0 ~ 161.8	162.5 153.4 ~ 171.3	156.0 154.0 ~ 161.0	163.8 155.8 ~ 174.8	157.0 154.1 ~ 166.5
BW (kg)	68.0 56.3 ~ 75.8	54.5 50.0 ~ 63.5	53.0 45.3 ~ 59.5	53.5 50.0 ~ 60.0	47.7 44.5 ~ 56.9	57.0 52.3 ~ 63.3	55.0 48.0 ~ 60.0	60.8 57.7 ~ 66.8	54.9 48.0 ~ 60.4

	BAS			NP			PMBV			PPMBV		
	CONT vs MOD	CONT vs HIGH	MOD vs HIGH	CONT vs MOD	CONT vs HIGH	MOD vs HIGH	CONT vs MOD	CONT vs HIGH	MOD vs HIGH	CONT vs MOD	CONT vs HIGH	MOD vs HIGH
Age	0.002	0.09	0.03	0.002		0.02	0.001		0.01	< 0.001		0.004
M\F		0.003			0.001	0.03		< 0.001	0.007		0.006	0.08
SR\AF	< 0.001	0.01	0.09	0.001	0.01		0.001	0.06	0.10	0.007	0.02	
Height					0.03			0.09				
BW		0.02	0.09		0.006	0.03		0.07			0.04	0.02

Number of patients in each cohort are in parentheses

Sex and heart rhythm are enumerated. For age, height and BW the upper value in each box is the median and the lower values are the 25th–75th percentiles. Statistical significance for sex and heart rhythm are assessed from 2 × 2 contingency tables by the Fisher exact test and for the other data by the Kruskal-Wallis test with Bonferroni correction. Only P values ≤ 0.10 are shown

*M\F* male\female, *SR\AF* sinus rhythm\atrial fibrillation, *BW* body weight, *BAS* baseline, *NP* nitroprusside, *PMBV* percutaneous mitral balloon valvuloplasty, *PPMBV* post-percutaneous mitral balloon valvuloplasty

### Calculations and Data Analysis

#### Standard Hemodynamics

In a previous publication [39], we described in detail the bulk of the calculations and data analysis methodology used herein. Hence, for the sake of brevity we provide an overview and details only as needed. We digitized the high-fidelity analog pressure and flow signals at a rate of 250 Hz (200 Hz for in those in AF) and analyzed them on a personal computer with a custom algorithm. We considered a beat acceptable only if the flow signal had no discernible baseline drift and no significant negative dip or secondary rise in diastole. We converted flow velocity to volume flow by multiplying the flow velocity signals averaged from several beats by the main pulmonary artery cross-sectional area. In the past we have found that using the scale factor built into the flowmeter hardware resulted in very inaccurate estimates of flow. Therefore, we applied a separate scaling factor to match the digitized volume flow to the estimated pulmonary flow obtained by using the patient’s estimated body surface area and oxygen consumption [40].

We have also found that, despite pre-soaking the high-fidelity pressure sensors, they still may have baseline drift. This drift is not only difficult to identify, but also can be large. Hence, we directly accounted for drift as follows. In the CONT we did not perform right heart catheterization prior to obtaining the high-fidelity data. Therefore we matched the diastolic pulmonary artery pressure measured by the Millar sensor to the left ventricular end-diastolic pressure obtained from the fluid-filled catheter [41]. We used the average of several beats to obtain the correction factor. In the MS patients we did likewise, but matched the mean pulmonary artery pressure (MPAP) from the Millar sensor to that obtained from the fluid-filled catheter. Additionally, even though it has been shown that quiet breathing has minimal effects on pulmonary impedance [26], to directly reduce the effect of respiratory variation we analyzed only those beats whose pressures at the start and end differed by ≤ 2 mmHg.

In addition to MPAP and systolic pulmonary artery pressure (SPAP) we also measured mean left atrial pressure (LAP) and mean right ventricular pressure (MRVP). We calculated pulmonary vascular resistance (PVR) and input resistance (R) as

1 $${\text{PVR }} = \, \left( {{\text{MPAP }}{-}{\text{ LAP}}} \right) \, /{\text{Q}} \, {\text{and}} \, {\text{R }} = {\text{ P}}_{0} /{\text{Q}}_{0}$$

where P_0_ and Q_0_ are the zeroth harmonics of the pressure and flow moduli, obtained as described below. In those cases in which we did not have LAP or pulmonary wedge pressure we used diastolic pulmonary artery pressure as an estimate of LAP [42]. At PPMBV, because LAP was also not available and because we included only patients whose mitral orifices had not restenosed, we used left ventricular end-diastolic pressure as an estimate of LAP.

We did not have LAP for all the conditions to estimate MVA using the standard formula [43, 44]. Therefore, for consistency, we estimated MVA for all conditions using the continuous-wave Doppler mode of the 2D echocardiogram. Briefly, we used the 2D mode with the apical 4-chamber view to identify the mitral orifice. Then we switched to the continuous-wave mode. Using auditory and visual monitoring, we angled the transducer so the beam showed maximal velocity across the valve. We made a hard-copy printout of the Doppler spectral density. From that printout we used the standard pressure half-time method to estimate MVA [45, 46]. After obtaining the data for estimating MVA, with the transducer in the same position, we switched to the color-Doppler mode and qualitatively assessed the amount of MR by visual inspection of the pseudo-color rendering.

We used data from an unpublished study from our laboratory to provide some assurance that our methodology provided reasonably accurate results. That study involved 77 patients with MS, including many from this study. In all those patients we had echocardiographic data as well as LAP measurements. The mean MVAs estimated by the standard formula from the LAP [43, 44] and those from the pressure half-time method were very close, 1.12 and 1.14 cm^2^, respectively. Additionally, linear regression of the MVA by these two methods revealed a strong correlation: R^2^ = 0.66, slope = 0.6, P < 0.001. The same team members made all the echocardiographic measurements in that and the present study. Hence, we have reasonable confidence in our methodology for estimating MVA in patients with MS

#### Impedance and Wave Reflections

We have previously described in detail how we obtain impedance parameters from the high-fidelity signals for patients in SR or AF [47]. Briefly, based on our experience [39, 47, 48], to get a representative view we averaged data from a minimum of six (mean, 7.63, SD 1.74, range, 6 to 19) acceptable beats. For patients in sinus rhythm (SR), we decomposed the pressure and flow into their Fourier harmonics. For each harmonic, the impedance modulus is the ratio of the pressure to flow harmonic. The phase angle is the difference of the pressure and flow phase angles.

For patients in atrial fibrillation (AF), we analyzed five data segments, each consisting of 9 consecutive seconds of data. We minimized leakage effects of any offset from the beginning to end of the segment by first removing the trend by linear interpolation and by multiplying with a Hanning window. We used a Fast Fourier Transform (FFT) algorithm to transform the time-domain signals into their frequency domain representations. This algorithm did not require 2^N^ data points so we did not zero pad the data. We calculated the impedance modulus, phase angle and coherence for each harmonic using standard spectral methods [49–52]. Briefly, we express the transformed pressure and flow signals for each data segment j in the frequency domain as P_j_(*f*,T) and Q_j_(*f*,T), respectively with *f* denoting frequency and T the duration of the segment. The one-sided autospectral density functions G_QQ_ and G_PP_ and the cross-spectral density function G_QP_ are:

2 $${\text{G}}_{{{\text{QQ}}}} = \, \left( {{2}/{\text{T}}} \right){\text{ E}}\left[ { \, \left| {{\text{Q}}_{{\text{j}}} \left( {f,{\text{T}}} \right)} \right|^{{2}} } \right] {\text{G}}_{{{\text{PP}}}} = \, \left( {{2}/{\text{T}}} \right){\text{ E}}\left[ { \, \left| {{\text{P}}_{{\text{j}}} \left( {f,{\text{T}}} \right)} \right|^{{2}} } \right] \;{\text{and}} \;{\text{G}}_{{{\text{QP}}}} = \, \left( {{2}/{\text{T}}} \right){\text{ E}}\left[ {{\text{Q}}_{{\text{j}}}^{*} \left( {f,{\text{T}}} \right){\text{P}}_{{\text{j}}} \left( {f,{\text{T}}} \right)} \right]$$

where * denotes complex conjugate, is the modulus of the complex number, and E is the expected value operator that averages over the segment j. The coherence at each frequency has a value between 0 and 1 and is

3 $${\text{C}}\left( f \right) \, = \, \left[ {\left| {{\text{ G}}_{{{\text{QP}}}} } \right|^{{2}} /{\text{G}}_{{{\text{PP}}}} {\text{G}}_{{{\text{QQ}}}} } \right]^{{{1}/{2}}}$$

The impedance modulus for each harmonic is

4 $${\text{Z}}(f) = {\text{ G}}_{{{\text{QP}}}} /{\text{G}}_{{{\text{QQ}}}}$$

and the impedance phase angle is the flow phase subtracted from the pressure phase. Retaining only those harmonics with coherence values > 0.9, we averaged the impedance moduli and phase angles from the five data segments. We further smoothed the results using a 9-point moving average.

In addition to R (Eq. 1), we calculated the following impedance parameters: total external power (W_t_); oscillatory power (W_o_); ratio of oscillatory to total power (W_o_/W_t_); first harmonic of impedance modulus (Z_1_); frequency of the first zero crossing of the impedance phase angle (F_0_); and characteristic impedance (Z_c_). The total external power is the sum of its mean and oscillatory terms, i.e.

5 $${\text{W}}_{{\text{t}}} = {\text{ W}}_{{\text{m}}} + {\text{ W}}_{{\text{o}}}$$

where

6 $${\text{W}}_{{\text{m}}} = {\text{ P}}_{0} {\text{Q}}_{0} \, {\text{and}} \, {\text{W}}_{{\text{o}}} = \sum\limits_{{{\text{i}} = 1}}^{{\text{n}}} {{\text{Q}}_{{\text{i}}}^{{2}} {\text{Z}}_{{\text{i}}} {\text{cos }}(\upphi_{{\text{i}}} )}$$

where i is the harmonic number, n is the highest harmonic and Q_i_, Z_i_ and ϕ_i_ are the flow, modulus and phase angle for harmonic i, respectively.

For patients in SR, we estimated Z_c_ by averaging the moduli harmonics beginning with frequencies higher than the first minimum of the modulus spectrum. We used Z_1_, to index the amplitude of the lower frequency portion of the impedance modulus spectra [53, 54]. For patients in AF, we estimated Z_c_ by averaging all impedance moduli between 12 and 50 Hz having a coherence > 0.9 [50–52]. We averaged the heart rate for all 5 data segments and used the modulus at that frequency to represent Z_1_. We estimated F_0_ by linearly interpolating the first zero crossing in the positive direction of the phase angle spectrum [32, 39].

Finally, we used the time-domain signals to assess wave reflections by decomposing the pressure and flow waves, P and Q, into their forward (P_for_ and Q_for_) and backward (P_back_ and Q_back_) components. We designate the amplitude of the pressure components as P_f_ and P_b_, respectively. The ratio P_b_ /P_f_ is an index of overall wave reflection magnitude [55]. Using this notation the measured pressure and flow waves, P and Q, are comprised of the sum of their forward and backward components as

7 $${\text{P }} = {\text{ P}}_{{{\text{for}}}} + {\text{ P}}_{{{\text{back}}}} \, {\text{and}} \, {\text{Q }} = {\text{ Q}}_{{{\text{for}}}} + {\text{ Q}}_{{{\text{back}}}}$$

Z_c_ relates the forward and backward components by the expressions

8 $${\text{P}}_{{{\text{for}}}} = {\text{ Z}}_{{\text{c}}} {\text{Q}}_{{{\text{for}}}} \, {\text{and}} \, {\text{P}}_{{{\text{back}}}} = \, - {\text{Z}}_{{\text{c}}} {\text{Q}}_{{{\text{back}}}}$$

Some algebraic manipulation results in the expressions for the forward and backward pressure components in terms of the measured waves as

9 $${\text{P}}_{{{\text{for}}}} = \, \left( {{\text{P }} + {\text{ Z}}_{{\text{c}}} {\text{Q}}} \right)/{2} \, {\text{and}} \, {\text{P}}_{{{\text{back}}}} = \, \left( {{\text{P }}{-}{\text{ Z}}_{{\text{c}}} {\text{Q}}} \right)/{2}$$

#### Right Ventricular Function

One way to assess right ventricular function is with the pump function approach which characterizes the relationship between mean ventricular pressure and output [56]. The ventricle operates on a function curve connecting the two axes. The intercepts on the abscissa and ordinate represent either a pure volume or pure pressure pump, respectively. Changes in load move the operating point along the function curve. In contrast, increases in contractility swivel the function curve up and to the right from its flow axis intercept. Increases in preload move the curve in parallel up and to the right.

### Statistical Analysis

For all statistical analyses, we used the SPSS statistical software. To compare categorical parameters, we tabulated 2 × 2 contingency tables and assessed statistical significance with the Fisher exact test. Because many parameters were not normally distributed, we used nonparametric tests. For patients with parameters that were repeated for two conditions, we used the Wilcoxon Signed Ranks test. Otherwise we used the Kruskal-Wallis test and applied the Bonferroni correction. As suggested recently [57, 58], rather than using the commonly accepted, but arbitrary, threshold of P < 0.05 to signify statistical significance, we report all P values ≤ 0.10 In addition, they suggest not to interpret results simply as significant or not significant. Rather, they recommend using the following terminology for different ranges of P values to describe whether or not there is a true effect differing from zero: *suggestion* of an effect (0.05 < P ≤ 0.10); *good evidence* of an effect (0.01 < P < 0.05); *strong evidence* of an effect (P < 0.01). We use their recommended terminology.

#### Discrete Analyses

**Fig. 1:**
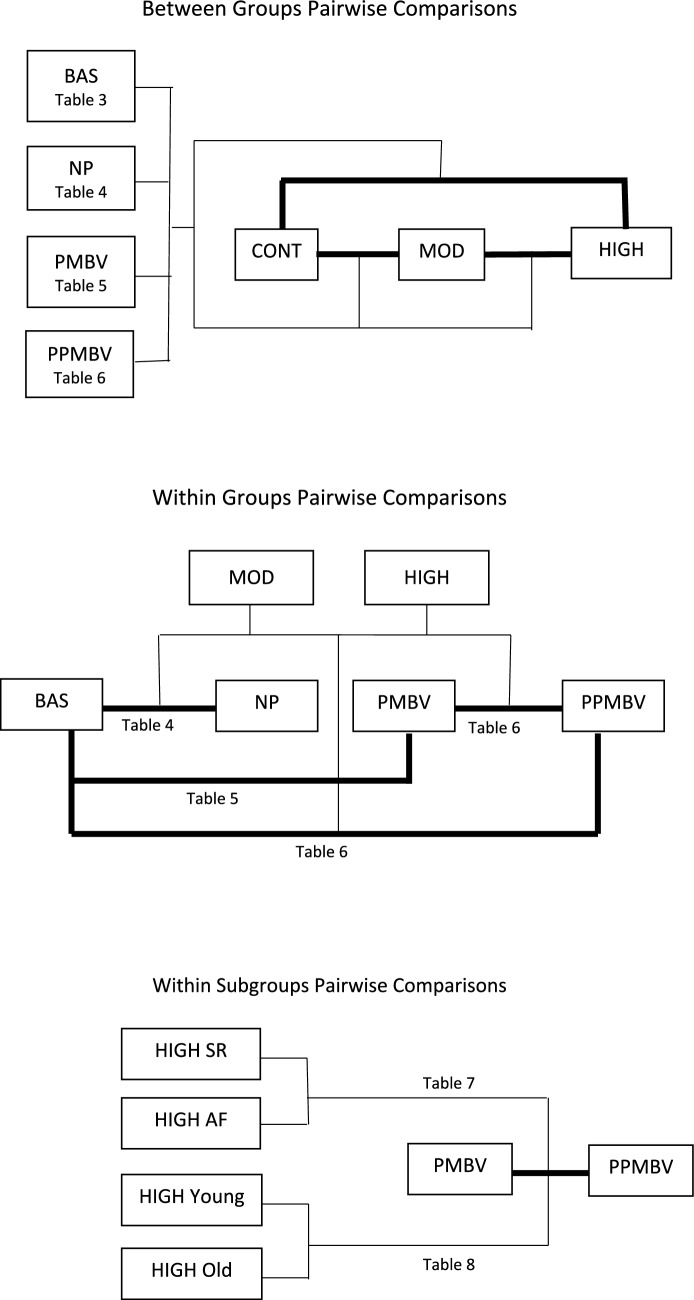
Flow charts of the discrete analyses procedures. The panels depict post-hoc pairwise comparisons: *between* main groups (top); *within* main groups (middle); and *within* subgroups (lower). The dark lines connect the pair of groups\conditions being compared. The light lines connect the group\condition for which the comparison is made. The tables identify the location of the statistical results. *CONT* control group, *MOD* mitral stenosis group with moderately elevated pulmonary pressure, *HIGH* mitral stenosis group with highly elevated pulmonary pressure, *SR* sinus rhythm subgroup, *AF* atrial fibrillation subgroup, *BAS* baseline, *NP* nitroprusside, *PMBV* percutaneous mitral balloon valvuloplasty, *PPMBV* post-percutaneous mitral balloon valvuloplasty

Treating pressure as a discrete variable, performing post-hoc pairwise comparisons of the parameters in the groups separated into pressure levels provides one perspective of pressure dependence. To distinguish this analysis from the regressions described below, we henceforth designate these as “discrete analyses”. The upper and middle panels of Fig. 1 are overviews of the *between* and *within* groups comparisons, respectively. The dark lines denote the pair of groups or conditions hypothesized to have no differences. The thin lines connect the condition or group to the pertinent pair being compared. We first determined which BAS parameters had *between* groups (MOD vs CONT, HIGH vs CONT and MOD vs HIGH) differences. To specifically assess whether the parameters for each MS group differed among the study conditions, without an influence from the CONT, we then omitted the CONT data and performed *within* group post-hoc pairwise comparisons for various pairs of conditions.

#### Regression Analyses

Treating pressure as a continuous variable, performing simple linear regression (SLR) with pressure as the predictor variable provides another perspective of pressure dependence. Accordingly, we performed SLR with MPAP as the predictor variable for many of the parameters. We excluded SPAP, MRVP and P_f_ because they are directly related to MPAP. We first compared the regressions with the MOD and HIGH data combined for each condition. To delineate the pressure dependence *between* and within groups for the different conditions, we then performed separate regressions for the MOD and HIGH for each condition.

## Results

### Overview

The upper portion of Table 1 summarizes some pertinent clinical and anthropometric data for the groups for each condition. The lower portion lists all P values ≤ 0.10 for the *between* groups pairwise comparisons for each condition. The numerous entries indicate substantial heterogeneity among the groups for all conditions. Specifically, there is good to strong evidence that the MOD is older than both the CONT and the HIGH for all conditions. There is also a suggestion that the HIGH BAS cohort is older than the CONT. The HIGH has a higher proportion of females than the CONT for all conditions and higher than MOD for all conditions except BAS. There is a different mixture of SR\AF among all the groups except between MOD vs HIGH at NP and PPMBV. The only differences in height are between the HIGH and CONT at NP and PMBV. The CONT have higher BW than the HIGH for all conditions. There is a suggestion that the BW of the MOD is higher than the HIGH for all conditions except PMBV.

**Fig. 2:**
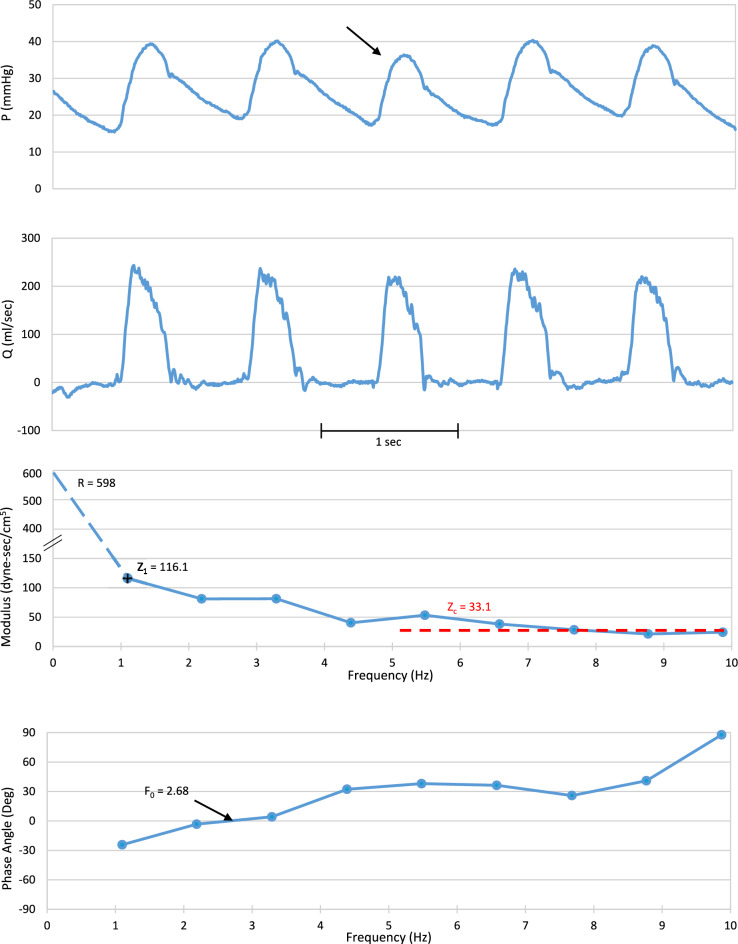
Representative digitized segment of simultaneously recorded pulmonary artery pressure (top) and flow (second from top) signals for a patient in sinus rhythm in the moderately elevated pulmonary artery pressure group. The two lower panels are the impedance modulus spectrum and phase angle spectrum obtained from standard Fourier series decomposition for the beat denoted. We “break” the modulus spectrum ordinate to enable visualization of both the intercept on the ordinate and sufficient detail in the higher frequency portion of the spectrum

**Fig. 3:**
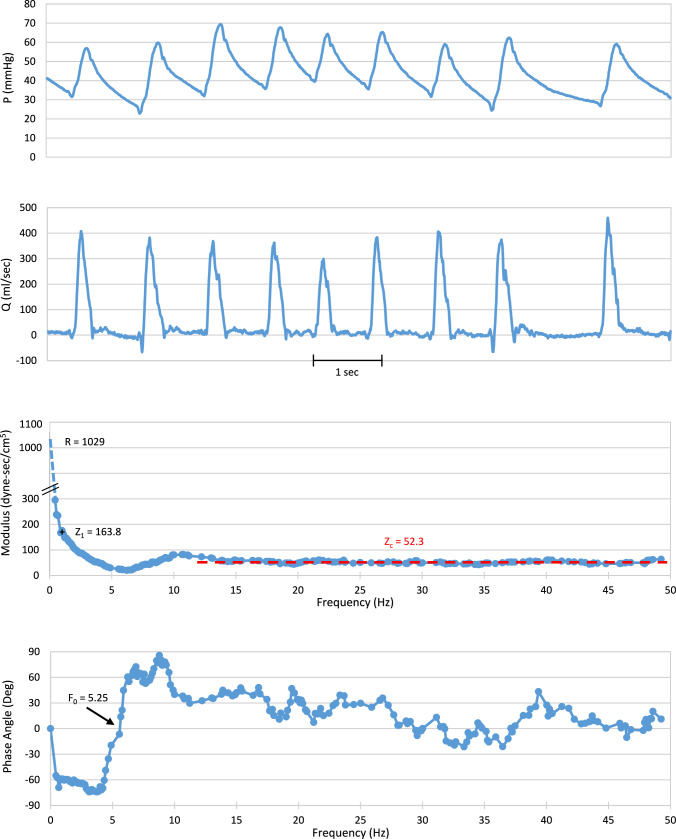
Representative digitized 9-s segment of simultaneously recorded pulmonary artery pressure (top) and flow (second from top) for a patient in atrial fibrillation in the highly elevated pulmonary artery pressure group. The two lower panels are the averaged impedance modulus and phase angle spectrum for five consecutive segments that include the segment shown. These spectra are obtained using standard spectral methods with an FFT algorithm (Eqs. 2–4). We “break” the modulus spectrum ordinate as explained in Fig. 2

The two upper panels in Fig. 2 show representative BAS digitized pressure and flow signals for a MOD patient in SR. The two lower panels show the impedance modulus and phase angle spectra for the denoted beat. Figure 3 shows a representative 9-s segment of the BAS digitized pressure and flow signals for a HIGH patient in AF. The lower panels show the averaged impedance modulus and phase angle spectra that included this data segment. For both the SR and AF, the modulus declines steeply from R at zero frequency to a minimum. At higher frequencies it oscillates above and below its asymptote which is Z_c_. For both the SR and AF spectra, in addition to R, we also denote two other key impedance parameters, Z_1_ and F_0_. To enhance visualization, we “break” the ordinates of the modulus spectra so as to both show R and to separate somewhat the higher frequency moduli of the different conditions.

**Fig. 4:**
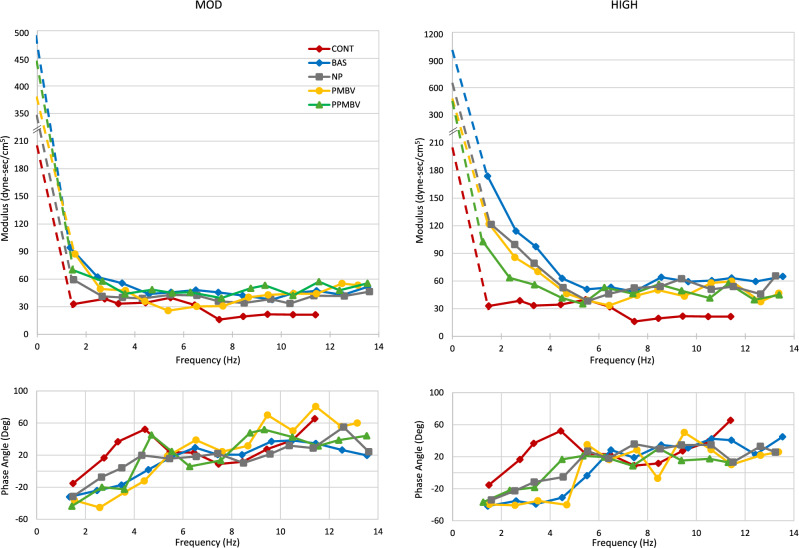
(Left panels) Impedance modulus (upper) and phase angle (lower) spectra for the control (CONT) group at baseline and different conditions for the cohorts with moderately (MOD) elevated pulmonary artery pressure. (Right panels) Analogous spectra for the cohorts with highly (HIGH) elevated pulmonary artery pressure. The legend denotes the study conditions for the cohorts of both groups. *BAS* baseline, *NP* nitroprusside, *PMBV* percutaneous mitral balloon vavluloplasty, *PPMBV* post-percutaneous mitral balloon valvuloplasty

The upper and lower panels in Fig. 4 show the aggregate impedance modulus and phase for the CONT and for each condition for the MOD and HIGH, respectively. To show the frequency dependence while still accommodating differing heart rates, we binned the data into 1-Hz intervals. Within each bin we show the averages for each group. As with Figs. 2 and 3, we “break” the ordinates of the modulus spectra. Although the spectra of patients in AF extend to higher frequencies than those shown, we only show the data in the frequency range of the patients in SR. Compared to CONT, the baseline R and lower frequency modulus spectrum of the MOD is shifted upward and the entire phase angle spectrum is shifted to higher frequencies. NP both decreases the low frequency portion of the modulus and shifts the phase to lower frequencies. Both PMBV and PPMBV have smaller effects than NP on the modulus and phase spectra. Compared to CONT, the HIGH has similar shifts in the modulus and phase spectra as the MOD, but both shifts are much larger. NP only partially reverses both these changes. Unlike in the MOD, however, in the HIGH there are progressive downward shifts from BAS to PMBV to PPMBV in the low frequency modulus spectra. In contrast, the leftward shift in the phase angle spectra appears to be larger for PPMBV compared to PMBV, with the latter being not as large as for NP. Regardless, the PPMBV results still differ from those of the CONT.

**Fig. 5:**
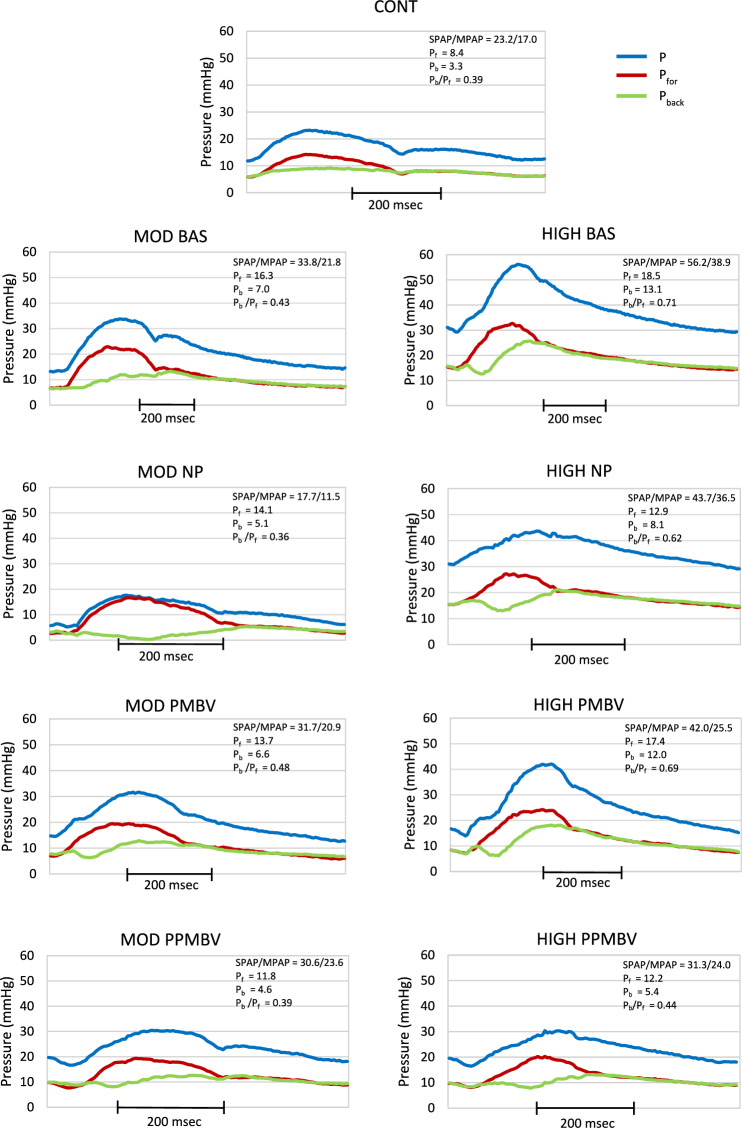
Representative beats showing the pulmonary artery pressure along with its forward (P_for_) and backward (P_back_) wave components as indicated in the legend. The top panel is for a patient from the CONT. The lower columns of panels are for the same patient, one from the moderately (MOD) and one from the highly (HIGH) elevated pulmonary artery pressure group for the conditions denoted in the panel titles. The insets list systolic and mean pressures along with the amplitudes of wave components, P_f_ and P_b_, as well as P_b_/P_f_ for the beat shown

Figure 5 shows representative single beats of P and its wave components for the groups and conditions. The insets list the values of SPAP, MPAP, P_f_, P_b_ and P_b_/P_f_. For each patient we selected a beat with values close to the median for that group. In the CONT P_back_ is low and broad resulting in P peaking in early systole and having a discernible “hump” after the dicrotic notch. The key findings in the MOD are the following:

- All the BAS pressures are higher and P_b_/P_f_ is slightly higher than the CONT. P_back_ rises from early systole and peaks in early diastole. This results in P peaking in systole later than in the CONT. There is still an early diastolic hump.
- NP decreases the pressure components resulting in P more closely resembling that of the CONT
- PMBV decreases the pressures and increases P_b_/P_f_ from their BAS values. P_back_ peaks earlier than at BAS resulting in P peaking in late systole with little or no diastolic hump.
- PPMBV decreases the pressure components and P_b_/P_f_ from BAS more than with PMBV causing the waveforms to closely resemble those of NP and the CONT.

The waveforms of the HIGH differ distinctly from those of the CONT and MOD as follows:

- The BAS pressures and P_b_/P_f_ are much higher than both the CONT and MOD BAS. P_back_ arrives and peaks early. This results in P having a clear inflection in early systole, a peak in late systole and no diastolic hump.
- NP decreases the pressures but they are still much higher than both the CONT and MOD NP.  P_back_ arrives later than at BAS resulting in P more closely resembling the CONT, albeit with higher values.
- PMBV only slightly decreases P_f_, P_b_ and P_b_/P_f_ from their BAS values. P_back_ is not otherwise affected so P more closely resembles that at BAS as opposed to NP.
- PPMBV produces further decreases in all the pressure components compared to PMBV. P_back_ is changed so that the P waveform more closely resembles those of NP and CONT.

### Discrete Analyses

Table 2 lists the median and 25th and 75th percentile values of the parameters for all groups and conditions. There are so many hemodynamic parameters that it is useful to categorize them as follows: pressures (LAP, SPAP, MPAP, MRVP), resistances (PVR and R), powers (W_t_, W_o_, W_o_/W_t_), impedances (Z_c_, Z_1_ and F_0_), and wave amplitudes (P_f_, P_b_, P_b_/P_f_). The aggregate results for the various groups and condition are detailed in the tables to follow. Because there are a large number of possible post-hoc pairwise comparisons, we show the pertinent statistical analyses results for the groups and conditions in the tables indicated in Fig. 1. To further help understand the large amount of data, we present the results for each condition separately.

**Table 2:** Parameter values for the control (CONT), moderately (MOD) and highly (HIGH) elevated pulmonary pressure mitral stenosis groups for the baseline (BAS), nitroprusside (NP), percutaneous mitral balloon valvuloplasty (PMBV) and post-percutaneous mitral balloon valvuloplasty (PPMBV) conditions

Parameter	CONT (n = 8)	MOD BAS (n = 21)	HIGH BAS (n = 33)	MOD NP (n = 19)	HIGH NP (n = 25)	MOD PMBV (n = 10)	HIGH PMBV (n = 15)	MOD PPMBV (n = 8)	HIGH PPMBV (n = 14)
PAD	2.17 1.89 ~ 2.62	2.15 2.00 ~ 2.45	2.27 1.95 ~ 2.87	NA	NA	2.14 1.99 ~ 2.41	2.20 2.10 ~ 2.95	1.97 1.70 ~ 2.00	2.13 1.84 ~ 2.37
MVA	NA	1.01 0.85 ~ 1.20	0.845 0.653 ~ 0.938	NA	NA	1.69 1.45 ~ 2.03	1.59 1.50 ~ 1.76	1.58 1.46 ~ 1.87	1.50 1.14 ~ 1.68
Q	101.6 78.7~133.3	57.1 51.7~66.4	56.9 43.3 ~ 64.2	61.7 45.6 ~ 73.3	60.6 45.3 ~ 76.2	70.6 58.3 ~ 94.8	78.4 51.1 ~ 96.2	69.3 65.4 ~ 83.4	69.2 51.8 ~ 83.3
LAP	10.5 8.30 ~ 11.0	19.0 16.0 ~ 20.0	24.5 19.8 ~ 29.5	10.6 7.56 ~ 15.6	21.4 17.3 ~ 28.4	10.0 7.75 ~ 14.5	11.5 7.00 ~ 21.5	12.0 10.0 ~ 14.8	13.0 9.00 ~ 14.3
SPAP	22.0 20.9 ~ 24.3	32.8 30.8 ~ 34.0	54.1 43.2 ~ 68.8	23.3 19.0 ~ 27.0	40.3 32.6 ~ 52.8	32.1 27.1 ~ 33.9	45.8 31.8 ~ 51.0	31.4 27.5 ~ 40.9	35.1 28.9 ~ 46.1
MPAP	15.4 13.1 ~ 17.4	23.0 20.0 ~ 25.0	39.0 33.0 ~ 49.0	16.2 12.5 ~ 21.2	32.4 25.2 ~ 39.2	20.0 19.0 ~ 23.3	33.0 21.0 ~ 38.0	22.5 19.3 ~ 28.8	24.5 19.8 ~ 29.8
MRVP	9.54 6.78 ~ 10.3	13.3 10.7 ~ 16.6	24.6 19.9 ~ 31.8	7.53 4.31 ~ 12.8	17.4 14.6 ~ 22.2	12.1 11.3 ~ 16.9	21.8 10.6 ~ 26.0	12.7 10.3 ~ 20.7	17.2 12.6 ~ 19.8
PVR	66.2 49.7~91.6	172.7 67.0~220.2	273.0 160.1~565.1	135.8 87.3~158.5	186.8 140.3~242.9	203.6 128.6~283.3	251.7 208.0~414.1	222.4 110.6~312.7	230.8 134.4~438.6
R	204.9 132.6 ~ 280.8	489.4 453.6 ~ 602.4	995.0 780.6 ~ 1309.6	349.3 229.3 ~ 475.3	651.5 538.4 ~ 801.8	382.9 292.2 ~ 518.8	476.7 353.1 ~ 754.1	441.1 352.9 ~ 480.5	450.6 338.9 ~ 757.2
Wt	328.5 281.8 ~ 365.7	211.9 164.5 ~ 253.9	374.9 239.9 ~ 469.5	165.9 118.9 ~ 232.2	289.6 213.8 ~ 417.0	265.0 213.8 ~ 337.8	414.1 280.2 ~ 490.8	361.2 257.7 ~ 392.1	307.7 205.1 ~ 398.1
W_o_	111.6 80.6 ~ 142.2	29.2 20.9 ~ 50.3	49.2 26.2 ~ 93.2	33.8 14.9 ~ 56.2	48.2 19.7 ~ 74.9	36.7 21.5 ~ 96.7	63.2 40.8 ~ 137.6	53.9 40.0 ~ 191.2	57.3 21.4 ~ 105.7
W_o_/W_t_	0.344 0.312 ~ 0.391	0.150 0.120 ~ 0.227	0.156 0.104 ~ 0.233	0.171 0.144 ~ 0.293	0.156 0.086 ~ 0.233	0.153 0.112 ~ 0.263	0.180 0.122 ~ 0.279	0.172 0.127 ~ 0.427	0.192 0.097 ~ 0.311
Z_c_	32.6 25.6 ~ 36.5	43.7 29.1 ~ 59.5	38.2 26.0 ~ 64.5	37.1 30.1 ~ 48.2	42.1 32.0 ~ 55.9	36.0 28.9 ~ 49.5	34.4 28.4 ~ 46.3	41.4 25.8 ~ 81.9	40.1 27.7 ~ 45.5
Z_1_	27.6 21.9 ~ 41.6	97.3 77.7 ~ 107.6	150.9 93.3 ~ 242.0	54.1 39.4 ~ 71.7	81.2 66.5 ~ 132.6	76.8 45.0 ~ 140.1	83.8 69.7 ~ 149.9	65.7 50.5 ~ 85.4	98.9 76.3 ~ 123.8
F_0_	2.19 1.77 ~ 2.34	4.49 3.24 ~ 5.34	5.25 4.70 ~ 5.92	3.21 2.28 ~ 4.15	4.53 3.20 ~ 5.32	5.06 4.26 ~ 5.82	4.97 4.39 ~ 5.45	2.98 2.54 ~ 4.34	4.12 2.84 ~ 4.69
P_f_	11.6 9.37 ~ 14.7	13.2 11.5 ~ 14.4	15.3 12.6 ~ 21.9	10.4 8.16 ~ 11.9	13.2 10.8 ~ 17.8	13.9 10.7 ~ 16.1	15.2 12.9 ~ 16.7	14.6 13.1 ~ 16.6	14.9 12.8 ~ 16.2
P_b_	4.92 3.24 ~ 6.66	6.15 5.44 ~ 7.89	9.99 7.16 ~ 17.6	4.45 3.17 ~ 5.50	6.84 5.03 ~ 10.9	7.05 5.74 ~ 8.65	8.02 6.44 ~ 13.2	7.32 6.58 ~ 9.84	6.42 5.53 ~ 10.1
P_b_/P_f_	0.420 0.339 ~ 0.476	0.498 0.392 ~ 0.600	0.687 0.594 ~ 0.784	0.425 0.319 ~ 0.593	0.544 0.450 ~ 0.708	0.517 0.480 ~ 0.601	0.618 0.500 ~ 0.782	0.533 0.462 ~ 0.687	0.498 0.438 ~ 0.639

In each box the upper value is the median and the values below are the 25th– 75th percentiles. NA = data not available. Number of patients in each cohort in parentheses

*PAD* pulmonary artery diameter (cm), *MVA* mitral valve area (cm^2^), *Q* pulmonary flow (ml/sec.), *LAP* mean left atrial pressure (mmHg), *SPAP/MPAP* systolic/mean pulmonary artery pressure (mmHg), *MRVP* mean right ventricular pressure (mmHg), *PVR* pulmonary vascular resistance (dyne-sec/cm^5^), *R* input resistance (dyne-sec/cm^5^), *W*_*t*_ total external power (mwatts), *W*_*o*_ oscillatory external power (mwatts), *Z*_*c*_ characteristic impedance (dyne-sec/cm^5^), *Z*_*1*_ first harmonic of impedance modulus (dyne-sec/cm^5^), *F*_*0*_ first zero-crossing of impedance phase angle (Hz), *P*_*f*_ magnitude of forward pressure wave component (mmHg), *P*_*b*_ magnitude of backward pressure wave component (mmHg)

#### BAS

Table 3 shows the results of the BAS *between* groups pairwise comparisons. The MVA of the HIGH differs from the MOD. We do not, however, have MVA data in the CONT to enable comparison with the MOD or HIGH. Compared to the CONT, in the MOD only a few parameters differ. Specifically, there is strong evidence of lower Q, the consequence of which are the lower powers. There is also good evidence for higher LAP, Z_1_ and F_0_ and a suggestion of a higher R. In contrast to the MOD, the HIGH has more extensive differences from the CONT. In fact, there is good to strong evidence that every parameter except Z_c_ and W_t_ differ. The substantial difference between the MOD and HIGH is highlighted in the last column which shows good to strong evidence of differences in every parameter except Q, Z_c_ and W_o_/W_t_.

**Table 3:** Baseline P values for the *between* groups pairwise posthoc comparisons for the CONT, MOD, and HIGH groups

Parameter	CONT vs MOD	CONT vs HIGH	MOD vs HIGH
PAD			
MVA	NA	NA	0.005
Q	0.001	< 0.001	
LAP	0.02	< 0.001	0.001
SPAP		< 0.001	< 0.001
MPAP		< 0.001	< 0.001
MRVP		< 0.001	< 0.001
PVR		0.001	0.03
R	0.07	< 0.001	< 0.001
W_t_	0.02		< 0.001
W_o_	0.001	0.03	0.04
W_o_/W_t_	0.001	< 0.001	
Z_c_			
Z_1_	0.01	< 0.001	0.001
F_0_	0.02	< 0.001	0.03
P_f_		0.03	0.01
P_b_		< 0.001	< 0.001
P_b_/P_f_		< 0.001	< 0.001

Statistical analyses performed using the Kruskal-Wallis test applying the Bonferroni correction. Only differences with P values ≤ 0.10 are listed. Abbreviations and units are defined in Table 2. NA indicates data not available

#### NP

Table 4 shows the results of the *between* and *within* groups analyses of the effects of NP. For the MOD the BAS vs NP column shows good to strong evidence that NP changes many parameters from their BAS values. Despite these clear effects, as shown in the CONT vs MOD column, differences between the MOD and CONT persist. Specifically, comparing the CONT vs MOD columns in Tables 3 and 4 reveals that NP only eliminates the BAS differences from CONT in LAP, R and F_0_. NP has different effects in the HIGH than in the MOD. The BAS vs NP column in Table 4 shows good evidence that NP lowers PVR and P_b_/P_f_ but, unlike in the MOD, does not affect LAP or W_t_. Similar to the MOD, after NP there continue to be differences in the HIGH vs CONT. In fact, comparing the CONT vs HIGH columns of Tables 3 and 4 indicates that NP only eliminates the difference of P_f_. NP also does not have a big effect on the differences between the MOD and HIGH. Specifically, comparing the MOD vs HIGH columns Tables 3 and 4 show that, among all the BAS parameters that differ, NP only eliminates the difference in W_o_.

**Table 4:** Left columns: P values for the *within* groups posthoc comparisons for the effects of NP versus BAS in the MOD and HIGH groups. Right columns: P values for the *between* group comparisons of CONT, MOD and HIGH

	Within groups		Between groups		
	MOD BAS vs NP	HIGH BAS vs NP	CONT vs MOD	CONT vs HIGH	MOD vs HIGH
PAD	NA	NA			
MVA	NA	NA	NA	NA	NA
Q			0.001	0.001	
LAP	< 0.001			< 0.001	< 0.001
SPAP	< 0.001	0.002		< 0.001	< 0.001
MPAP	< 0.001	0.002		< 0.001	< 0.001
MRVP	0.001	0.002		0.005	< 0.001
PVR		0.05		< 0.001	0.002
R	0.002	0.002		< 0.001	< 0.001
W_t_	0.03		< 0.001		< 0.001
W_o_			< 0.001	0.003	
W_o_/W_t_			0.02	< 0.001	
Z_c_					
Z_1_	< 0.001	0.01	0.10	< 0.001	< 0.001
F_0_	0.02	0.03		< 0.001	0.008
P_f_	0.001	0.05			0.002
P_b_	0.003	0.007		0.08	0.001
P_b_/P_f_		0.007		0.03	0.009

Statistical analyses performed using the Kruskal-Wallis test applying the Bonferroni correction. Only differences with P values ≤ 0.10 are listed. Abbreviations and units are defined in Table 2. NA indicates data not available.

#### PMBV

Table 5 shows the results of PMBV. As shown in the *within* group columns, MVA is increased from its BAS values in both groups. This eliminates the BAS difference between them. Despite this anatomic effect, however, the hemodynamic effects are modest. The only effects are decreases in LAP, Q and R in both groups and decreases in MPAP and Z_1_ in the HIGH. Comparing the corresponding *between* groups columns in Tables 3 and 5 reveals a limited effect. In both the MOD and HIGH, PMBV eliminates the BAS differences from the CONT in LAP. In the MOD it also eliminates the differences in Q and W_t_. There is no effect in either the MOD or HIGH on their BAS differences from CONT in other parameters. Comparing the MOD vs HIGH columns of Tables 3 and 5 reveals that, among all the parameters that differ at BAS, only the differences in SPAP and MPAP are eliminated.

**Table 5:** Left columns: P values for the *within* groups posthoc comparisons for the effects of PMBV versus BAS in the MOD and HIGH groups. Right columns: P values for the *between* group comparisons of CONT, MOD and HIGH.

	Within groups		Between groups		
	MOD BAS vs PMBV	HIGH BAS vs PMBV	CONT vs MOD	CONT vs HIGH	MOD vs HIGH
PAD					
MVA	< 0.001	< 0.001	NA	NA	
Q	0.06	0.02		0.06	
LAP	< 0.001	< 0.001			
SPAP			0.04	< 0.001	0.05
MPAP		0.06	0.06	< 0.001	0.05
MRVP			0.07	0.003	
PVR			0.02	< 0.001	
R	0.07	0.001	0.05	< 0.001	
Wt					
W_o_			0.05		
W_o_/W_t_			0.007	0.007	
Z_c_					
Z_1_		0.06	0.01	< 0.001	
F_0_			0.001	< 0.001	
P_f_					
P_b_				0.006	
P_b_/P_f_			0.09	0.001	

Statistical analyses performed using the Kruskal-Wallis test applying the Bonferroni correction. Only differences with P values ≤ 0.10 are listed. Abbreviations and units are defined in Table 2. NA indicates data not available.

#### PPMBV

Table 6 shows the results of PPMBV. The PPMBV vs PMBV columns show evidence for a change in F_0_ in both the MOD and HIGH. The BAS vs PPMBV columns show, however, that F_0_ still differs from its BAS value in the HIGH but not the MOD. Regardless, the *between* groups columns suggest that F_0_ still differs from CONT in both the MOD and HIGH. As for other parameters, compared to their BAS values, PPMBV changes a few in the MOD but more in the HIGH. As a result, as shown in the MOD vs HIGH columns of Tables 3 and 6, the BAS differences between the MOD and HIGH of all parameters except Z_1_ are eliminated. Nevertheless, as shown in the CONT vs MOD and CONT vs HIGH columns, many parameters in both groups, as with F_0_, still differ from those of the CONT.

**Table 6:** Left columns: P values for the *within* groups posthoc comparisons for the effects at PPMBV versus BAS in the MOD and HIGH groups. Right columns: P values for the *between* group comparisons of control CONT, MOD and HIGH

	Within groups				Between groups		
	MOD		HIGH				
	BAS vs PPMBV	PPMBV vs PMBV	BAS vs PPMBV	PPMBV vs PMBV	CONT vs MOD	CONT vs HIGH	MOD vs HIGH
PAD	0.08						
MVA	0.003		< 0.001		NA	NA	
Q	0.02				0.08	0.005	
LAP	0.003		< 0.001				
SPAP			0.001		0.02	0.001	
MPAP			< 0.001		0.01	0.001	
MRVP			0.002		0.04	0.003	
PVR					0.04	0.002	
R			< 0.001		0.02	0.001	
Wt	0.009						
W_o_						0.06	
W_o_/W_t_						0.05	
Z_c_							
Z_1_	0.09		0.05		0.07	< 0.001	0.02
F_0_		0.02	0.004	0.08	0.06	0.003	
P_f_							
P_b_			0.03		0.01	0.08	
P_b_/P_f_			0.007		0.06	0.10	

Statistical analyses performed using the Kruskal-Wallis test applying the Bonferroni correction. Only differences with P values ≤ 0.10 are listed. Abbreviations and units are defined in Table 2. NA indicates data not available.

### Subgroup Discrete Analyses

Because there are effects at PPMBV compared to PMBV, we performed additional analyses to explore the role of age and cardiac rhythm. The overview for these subgroup analyses and the pertinent results tables are shown in the bottom chart of Fig. 1. Unfortunately, the number of patients in the MOD are insufficient to enable robust statistical analyses, so we limit this additional analyses to the HIGH. To explore the role of cardiac rhythm, we first divided the HIGH into SR and AF subgroups and compared the conditions *within* each subgroup, focusing only on the PMBV vs PPMBV comparison. Analogously, we then subdivided the HIGH into old and young subgroups, using 43 years as the dividing age. Table 7 shows the results of the rhythm subgroup analyses. The differences between the subgroups in a few parameters are indicated by the asterisks. In particular, there is some evidence for a lower flow in the AF compared to SR subgroup at BAS and PPMBV. This is manifested in the lower powers and higher resistances, particularly after PPMBV. The PPMBV vs PMBV column in the SR subgroup reveals a suggestion of a difference for W_o_/W_t_, some evidence for a difference for PVR and very strong evidence for a difference for F_0_. In contrast, there are no PPMBV vs PMBV differences in the AF subgroup. Table 8 shows analogous results for the age subgroups. Compared to the rhythm subgroups there are fewer parameters that differ. Most interestingly, the PMBV vs PPMBV comparison reveals good evidence for differences for the pressures, W_t_ and F_0_ in the young subgroup and weaker evidence of a difference for F_0_ in the old subgroup.

**Table 7:** Results for the HIGH sinus rhythm (SR) and atrial fibrillation (AF) subgroups for the BAS, NP, PMBV and PPMBV conditions

Parameter	HIGH SR				
	BAS (16)	NP (12)	PMBV (9)	PPMBV (6)	PMBV vs PPMBV
MVA	0.85		1.70	1.58	
Q	60.3	62.0	81.0	84.7	
LAP	24.0	26.2	9.00	13.0	
SPAP	56.1	43.1	47.1	30.8	
MPAP	44.0	33.3	33.0	20.5	
MRVP	28.3	18.0	24.6	14.6	
PVR	337.7	168.1	261.6	147.4	0.05
R	998.1	600.8	455.2	334.1	
W_t_	451.7	337.8	442.4	338.6	
W_o_	86.8	71.2	114.2	107.0	
W_o_/W_t_	0.23	0.21	0.27	0.32	0.10
Z_c_	35.9	38.7	34.1	37.7	
Z_1_	152.1	90.9	93.4	78.6	
F_0_	5.00	3.80	5.33	3.04	0.007
P_f_	14.3	11.2	15.6	14.9	
P_b_	10.4	6.82	10.0	6.56	
P_b_/P_f_	0.72	0.53	0.66	0.46	

Parameter	HIGH AF				
	BAS (17)	NP (13)	PMBV (6)	PPMBV (8)	PMBV vs PPMBV
MVA	0.83		1.55	1.44	
Q	50.3*	55.3	66.3	61.5^	
LAP	27.0	20.1	19.5	12.0	
SPAP	52.0	40.3	42.6	39.8	
MPAP	39.0	27.3	31.5	28.0	
MRVP	23.8	16.7	21.4	19.3	
PVR	272.5	186.8	247.6	381.2*	
R	964.8	673.3	573.8	678.2^	
W_t_	258.0^#^	232.7	315.6*	220.6*	
W_o_	26.6^#^	20.0^#^	38.9^#^	25.3^#^	
W_o_/W_t_	0.10^#^	0.09^#^	0.12^#^	0.11^#^	
Z_c_	49.8	42.7	39.5	40.8	
Z_1_	149.6	81.2	81.0	111.5	
F_0_	5.40	4.92^	4.68	4.34*	
P_f_	16.0	14.3	14.3	14.9	
P_b_	9.42	6.84	7.78	6.09	
P_b_/P_f_	0.67	0.54	0.56	0.57	

Only P values ≤ 0.10 are listed for the *within* subgroup (PMBV vs PPMBV) pairwise comparisons. Number of patients in each cohort in parentheses

For clarity only the median values of the parameters are shown.

The Kruskal-Wallis test with the Bonferroni correction used for all pairwise comparisons.

*P < 0.10, ^P < 0.05, ^#^P < 0.005 for SR vs AF *between* subgroups comparisons.

Abbreviations and units are defined in Table 2.

**Table 8:** Results for the HIGH Young and Old subgroups for the BAS, NP, PMBV and PPMBV conditions

Parameter	HIGH Young				
	BAS (15)	NP (12)	PMBV (7)	PPMBV (7)	PMBV vs PPMBV
MVA	0.83		1.60	1.50	
Q	58.3	66.2	88.7	74.3	
LAP	25.0	24.8	18.5	13.0	
SPAP	54.8	43.1	47.1	29.8	0.03
MPAP	41.0	33.1	33.0	20.0	0.01
MRVP	20.8	17.0	24.6	13.5	0.007
PVR	197.5	190.1	218.1	148.4	
R	995.0	579.9	464.4	362.9	
W_t_	407.5	320.8	421.7	306.5	0.06
W_o_	74.7	59.3	114.2	84.6	
W_o_/_Wt_	0.17	0.17	0.18	0.28	
Z_c_	37.5	38.9	34.1	35.1	
Z_1_	140.1	78.6	69.7	80.6	
F_0_	5.23	4.40	5.24	3.07	0.02
P_f_	14.5	13.7	15.6	15.2	
P_b_	9.42	6.82	8.02	6.92	
P_b_/P_f_	0.64	0.53	0.64	0.54	

Parameter	HIGH Old				
	BAS (18)	NP (13)	PMBV (8)	PPMBV (7)	PMBV vs PPMBV
MVA	0.86		1.58	1.52	
Q	50.3	50.8	65.9*	68.8	
LAP	22.0	20.2	7.50	13.0	
SPAP	52.8	40.3	40.9	38.2	
MPAP	39.0	29.1	27.0	28.0	
MRVP	24.6	18.3	12.7	18.7	
PVR	305.3	177.5	278.1	379.7	
R	996.9	673.3	573.8	654.0	
W_t_	333.2	232.7	289.3*	308.8	
W_o_	39.3	32.7^	45.6	44.6	
W_o_/_Wt_	0.12	0.11	0.17	0.16	
Z_c_	51.1	42.3	44.7	40.6	
Z_1_	155.4	99.2	96.4*	113.0	
F_0_	5.33	4.67	4.85	4.29	0.08
P_f_	16.0	13.2	14.3	14.5	
P_b_	9.99	6.84	7.80	6.20	
P_b_/P_f_	0.71	0.57	0.59	0.46	

Only P values ≤ 0.10 are listed for the *within* subgroup (PMBV vs PPMBV) pairwise comparisons. Number of patients in each cohort in parentheses

For clarity only the median values of the parameters are shown.

The Kruskal-Wallis test with the Bonferroni correction used for all pairwise comparisons.

*P < 0.10, ^P < 0.05 for Young vs Old between subgroup comparisons.

Abbreviations and units are defined in Table 2

### Right Ventricular Function

**Fig. 6:**
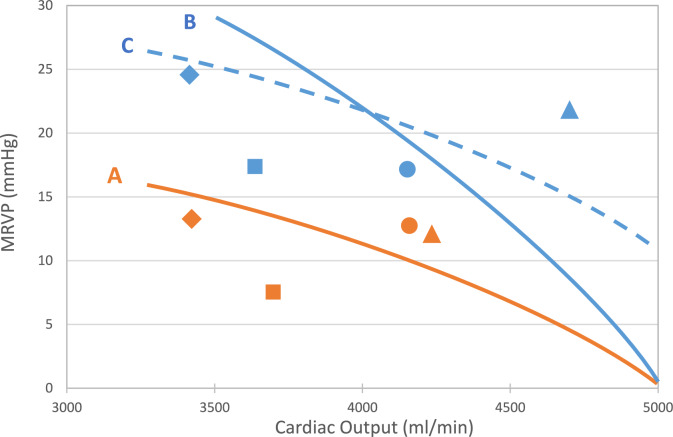
Scatterplot of mean right ventricular pressure as a function of cardiac output for the moderately elevated (MOD) and the highly elevated (HIGH) pulmonary artery pressure group at BAS (Δ); during NP (◊), at PMBV (□) and at PPMBV (○). The orange and blue symbols represent the MOD and HIGH, respectively. The symbols are the medians for each group\condition. Curve A is a conceptualization of a pump function curve that fits the MOD data reasonably well. Curves B and C are depictions of pump function curves with increased contractility or preload, respectively, from Curve A. Either curve fits the HIGH data reasonably well. The curves are not meant to include the NP data

Figure 6 is a scatterplot of the MRVP and cardiac output for the MS groups for the various conditions. Even though there are insufficient data to reliably depict the function curves, for illustrative purposes, we include reasonable conceptualized curves. Curve A depicts a function curve that fits the MOD data (without the NP datum). Curve B depicts a function curve with increased contractility from the MOD. Curve C represents a function curve with increased preload from the MOD. The HIGH data, also excluding NP,  seem to fit either curves B or C.

### Regression Analyses

**Fig. 7:**
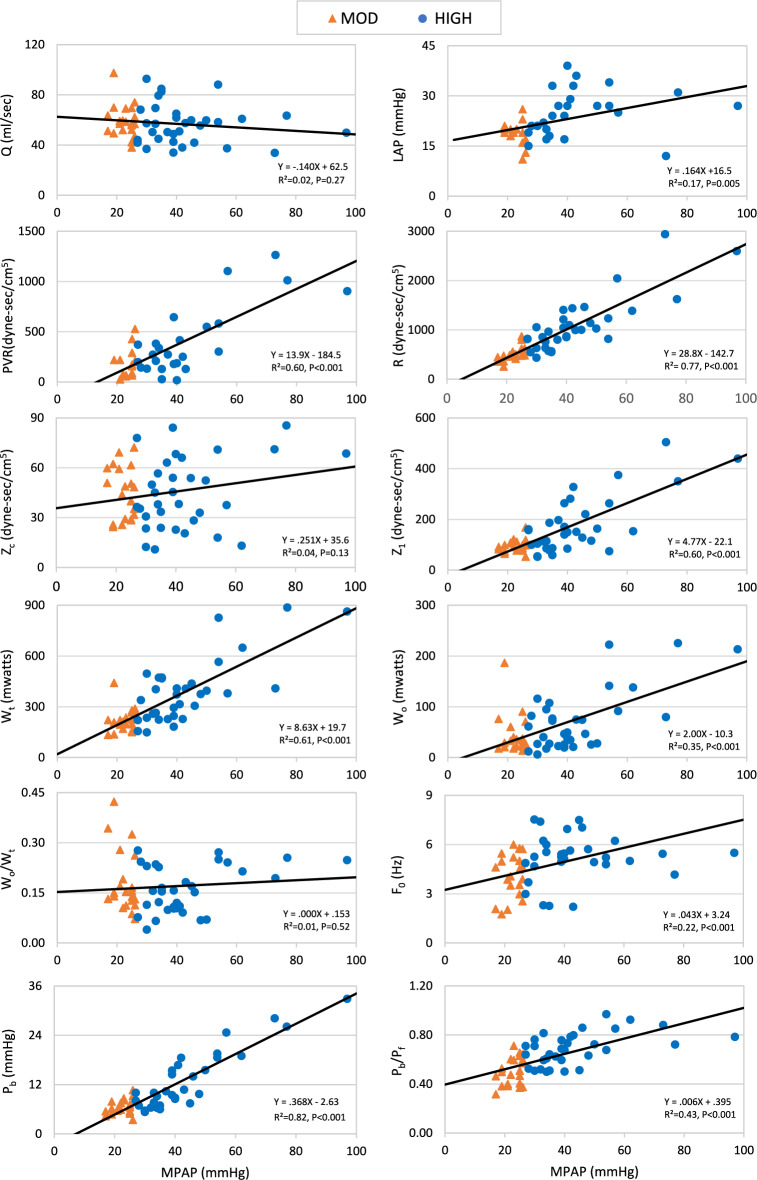
Scatterplots of the hemodynamic parameters with mean pulmonary artery pressure (MPAP) as the predictor variable during baseline conditions. The symbols are the median values for the moderately (MOD) and highly (HIGH) elevated pulmonary artery pressure groups. For ease of identification, we use the same colors as in Fig. 6 and the same symbols as in Fig. 4. The linear regression results and coefficients are in the insets. *Q* pulmonary flow, *LAP* left atrial pressure, *R* input resistance, *PVR* pulmonary vascular resistance, *Z*_*c*_ characteristic impedance, *Z*_*1*_ magnitude of first harmonic of impedance modulus, *W*_*t*_ total external power, *W*_*o*_ oscillatory power, *F*_*0*_ first zero crossing of impedance phase angle, *P*_*b*_ magnitude of backward pressure wave, *P*_*b*_*/P*_*f*_ wave reflection index

The SLR results for the combined MOD and HIGH at BAS are shown in Fig. 7. Eight of the 12 parameters have a strong, significant dependence on MPAP. LAP has a weak dependence and Q, W_0_/W_t_ and Z_c_ do not depend upon MPAP.

Table 9 shows the results of the separate regression analyses. For the *between* groups comparisons in the upper half of the table, there are many more entries in the HIGH compared to the MOD columns for BAS, NP and PMBV. This indicates that the HIGH is primarily responsible for the strong dependence on MPAP seen with the combined MOD and HIGH data. At PPMBV the MOD and HIGH have fewer differences. Regardless, as shown by the sparse number of P values in the MOD vs HIGH columns, very few of the parameters have a different pressure dependence between the groups. Only with NP is there good or strong evidence of a difference. Similarly, the sparse number of entries in the lower half indicate very few parameters have a difference in their pressure dependence between any pair of conditions.

**Table 9:** Results of the *between* and *within* groups separate regressions for the hemodynamic parameters with a strong dependence on MPAP for the MOD and HIGH groups and conditions

	BAS			NP			PMBV			PPMBV		
	MOD	HIGH	MOD vs HIGH	MOD	HIGH	MOD vs HIGH	MOD	HIGH	MOD vs HIGH	MOD	HIGH	MOD vs HIGH
PVR		< 0.001		0.04	< 0.001		0.004	0.002		< 0.001	< 0.001	
R	0.001	< 0.001		< 0.001	< 0.001		0.03	< 0.001		0.001	0.002	
W_t_		< 0.001		0.03	0.007			0.05				
W_o_		< 0.001	0.07		0.09				0.08			
Z_1_		< 0.001			< 0.001	0.04		< 0.001		0.003	0.02	
F_0_		0.02			0.09			0.03			0.02	
P_b_		< 0.001			< 0.001	0.007	0.06	0.002		0.08		
P_b_/P_f_	0.08	0.003			0.007			0.05				

	MOD				HIGH			
	BAS vs NP	BAS vs PMBV	BAS vs PPMBV	PMBV vs PPMBV	BAS vs NP	BAS vs PMBV	BAS vs PPMBV	PMBV vs PPMBV
PVR								
R								
W_t_								
W_o_							0.01	
Z_1_								0.09
F_0_								
P_b_							0.02	
P_b_/P_f_								

Upper portion: P values of the regression slopes and *between* groups pairwise comparisons for the BAS, NP, PMBV and PPMBV conditions. Lower portion: P values of the *within* groups pairwise comparisons for the MOD and HIGH groups. Only P values ≤ 0.10 are listed

Statistical analyses performed using the Kruskal-Wallis test applying the Bonferroni correction

Abbreviations and units are defined in Table 2

## Discussion

### Context

#### BAS

Our baseline standard hemodynamic findings are generally consistent with previous reports in patients with MS. Specifically, the PVR in the HIGH of 273 dyne-sec/cm^5^ is in the range of 240 to 400 dyne-sec/cm^5^ previously reported in MS patients with MPAP levels similar to ours [9, 12–14, 19, 20, 31–33]. Additionally, reports of higher PVR values in patients with higher MPAP [11, 14, 31] are consistent with our and previous findings [25] of a strong linear association between MPAP and PVR. With respect to pulsatile hemodynamics, our findings of higher R, Z_1_, P_b_/P_f_, and F_0_ compared to the CONT are consistent with the limited data available [30–33]. The lower W_t_ we found in the MOD vs CONT was also previously reported [31] but differs from the increase found in an early study [32]. Likewise, the lower than normal W_o_/W_t_ we found is consistent with that reported in [32] but differs from the apparent lack of difference reported in [31]. We do not know the reasons for these different findings.

Our finding that Z_c_ in the MS groups did not differ from the CONT in any condition differs from previous findings. The earliest study involving 3 normal and 7 MS patients [32] found that, among the 4 lowest Z_c_ values, 3 were in normal patients. Statistical analysis could not be performed, however, to see if the difference between normal and MS was significant. In three later studies Z_c_ was stated or appeared to be higher than normal [30, 31, 33]. This discrepancy with our finding is likely due to a methodological error in those studies. Z_c_ in patients in SR is correctly estimated by averaging the harmonics of the impedance modulus beyond its first minimum. Because the modulus spectrum falls steeply in the frequency range from zero to its first minimum, including harmonics in the steeply falling range will overestimate Z_c_. Recognizing this, the first study [32] recommended that averaging should begin at 5 Hz rather than the standard 2 Hz. This is because the frequency of the first minimum of modulus in MS is shifted to higher frequencies than normal. All three later studies, however, began averaging at or near 2 Hz despite the first minimum being shifted to higher frequencies. Therefore, it is a near certainty that those estimates of Z_c_ are erroneous.

We performed additional analyses to directly demonstrate the impact of this error. We reanalyzed our BAS data only in those in SR by estimating Z_c_ beginning at 2 Hz. For clarity we call this re-estimated parameter Z_c_*. For those in AF, estimating Z_c_ is very reliable. This is because, as shown in Fig. 3, many more harmonics are available and applying the coherence criterion allows robust estimates at higher frequencies. Despite the SR patients being a minority in both groups, the reanalyzed results revealed evidence for differences in Z_c_* in both MOD vs CONT (P = 0.06) and HIGH vs CONT (P = 0.01). In contrast, Z_c_ and Z_c_*did not differ in the CONT. Hence, incorrectly estimating Z_c_, as was done in those earlier studies, would have changed one of our major conclusions.

#### PMBV

Immediately after PMBV our finding that PVR in both the MOD and HIGH still differs from the CONT is generally consistent with previous reports [11, 12, 14, 18–20, 31]. Three studies, however, reported significant decreases in PVR immediately after valvuloplasty [9, 13, 17]. We do not know the reason for these discrepant results. Like previously reported [31], we found that PMBV in the HIGH decreased Z_1_. However, our finding in the HIGH of no effect on the power terms or P_b_/P_f_ differs from the significant decreases in W_o_/W_t_ and reflection factor they reported. One possible explanation for these different findings is the larger decrease in MPAP (37 to 27 mmHg) compared to ours (39 to 33 mmHg). Another possibility relates to the issue with Z_c_ discussed earlier since it affects both P_for_ and P_back_.

#### PPMBV

Our findings of further changes in the vasculature at PPMBV are consistent with previous follow-up studies suggesting some reverse remodeling [11, 12, 18, 20]. Of note are two studies which reported that the decreases in MPAP and PVR occurred within 6 to 24 h after PMBV [9, 19]. In contrast, however, a large study of 244 patients showed no further decrease in resistance index at 12 month follow-up [17]. There are, however, no reports of the short-term effects of PPMBV on pulsatile hemodynamics or wave reflections with which to directly compare our results.

The subset analyses in the HIGH suggest that SR and/or young age are factors that favor reverse remodeling after PMBV. There are other studies that implicate either heart rhythm and/or age as factors in remodeling and or long-term outcomes after PMBV. One study found that MPAP and PVR did not normalize immediately after PMBV but did so 12 months afterward [12]. Twenty of the 21 patients in that study were in SR and the mean age of the patients was 27 years. Several other larger studies with long follow-up times also found that older age and/or AF were predictive factors for poor outcomes [10, 16, 59, 60]. Another study involving 30 young adults with MS, all of whom were in SR, showed that left atrial p wave dispersion and electromechanical delay, two harbingers of future development of AF, were decreased shortly after PMBV [61]. The potential roles of age and AF are further underscored by a large study on the long-term effects of mitral valve replacement [62]. With patients segregated into low and high mitral valve pressure gradient groups, the low gradient group, which was older and had more patients in AF, had less favorable LA remodeling, more impairment of LV diastolic function and a greater risk for persistent symptoms. Another study assessed left atrial anatomy and function at 72 h and 12 months after PMBV [63]. At 72 h, the increase in MVA and decreases in MPAP and LAP were associated with increases in LA ejection and decreases in LA dimensions. These changes were sustained over 12 months. They attributed these changes to LA remodeling. Unfortunately, it is not clear whether the changes at 72 h were a passive effect of the PMBV or actual remodeling.

### Implications

#### Pressure Dependence

Our SLR regression results indicate that many parameters have a strong dependence on MPAP over the range spanned by our patients. Moreover, the separate regression analyses results suggest that neither PMBV nor PPMBV changed the pressure dependence of the vast majority of the parameters. In contrast, as shown in Table 9, NP changed the pressure dependence of Z_1_ and P_b_ in the MOD compared to the HIGH. The regression analyses provide, however, only one perspective on pressure dependence. The discrete analyses of results grouped by different pressure levels provide different and more quantitative information. Differences between the MOD and HIGH in any parameter for any condition is, in fact, evidence for pressure dependence. Therefore, our results provide clear evidence for pressure dependence of most parameters. Our results do not, however, enable us to delineate the underlying mechanism(s) of the higher MPAP in the HIGH vs the MOD. Severity of disease, as indexed by MVA, could play a role as it was smaller in the HIGH than the MOD. However, the separate regression results indicating no difference in dependence on MPAP of MVA between the MOD and HIGH argues against MVA being an important factor. Age is an indirect indicator of duration of disease. Duration, however, is not likely to be the factor since the MOD were older than the HIGH.

#### Impedance and Wave Reflections

Our results for the key impedance parameters, Z_1_, F_0_ and P_b_/P_f_ are indicative of substantial alterations in the vasculature due to MS. The higher BAS levels of Z_1_ in both groups compared to the CONT are indicative of increased pulsatile ventricular loading. The findings that they are still higher than in the CONT after both PMBV and PPMBV show that the vasculature is not returned to its control state. Therefore the ventricle continues to face excess loading.

The alterations from the CONT in both F_0_ and P_b_/P_f_ indicate altered wave reflection properties. As shown in Fig. 5, the pressure wave components are major determinants of the amplitude and shape of the measured pressure. Compared to CONT P_b_/P_f_ is elevated, particularly in the HIGH BAS. After both PMBV and PPMBV it remains higher than in the CONT. This persistent alteration in the wave reflection index is also borne out by the results for F_0_.

An elevated F_0_ can be due to either increased wavespeed and/or a more proximal major wave reflection site [52]. Wavespeed is directly related to vascular stiffness which can be either actively increased via vasoconstriction or by passive distention [50, 54, 64]. F_0_ in the HIGH did not decrease appreciably after PMBV, despite a large decrease in MPAP, which would decrease distention and hence wavespeed. This suggests that a more proximal reflection site rather than increased wavespeed is primarily responsible for the elevated F_0_. Furthermore, the finding in the MOD that PPMBV decreased F_0_ nearly to the CONT level without much further decease in MPAP also suggests an altered reflection site. Additionally, the finding in the MOD that F_0_ no longer differed from the CONT after NP suggests that vasoconstriction plays some role. Further support for this was the finding in the HIGH that F_0_ was decreased by NP. However, since PPMBV decreased F_0_ from its BAS level, but not to CONT levels, we cannot rule out the possibility that PPMBV also decreased some vasoconstriction. Thus, both the magnitude of wave reflections and the major reflection site are affected by MS.

One noteworthy finding is that Z_c_ did not differ from the CONT in either the MOD or HIGH. Z_c_ is directly related to vascular stiffness and inversely related to size of the most proximal portion of the vasculature. The baseline Z_c_ in both the MOD and HIGH, despite higher than normal distending pressures, did not differ from that of the CONT. This suggests that any stiffness increase due to vasoconstriction was counterbalanced by the passive effects of vessel distention. In the MOD, because of the small effect of PMBV on MPAP, the unchanged Z_c_ suggests that PMBV did not discernibly decrease vasoconstriction, otherwise Z_c_ should have decreased. In the HIGH the situation is more complex. In pulmonary arteries which have nonlinear stress-strain properties [65], at high distending pressures the vessel is close to its limit of distension. Consequently, the large decrease in MPAP and the subsequent effect of the passive decrease in stiffness likely outweighed the effect of a smaller vessel. The unchanged Z_c_ suggests that there was not a major effect on vasoconstriction, otherwise the combined passive decrease in stiffness and decreased vasoconstriction should have decreased Z_c_.

#### Clinical Perspective

Besides providing detailed insight into hemodynamics, impedance and wave reflections also have clinical implications. The results shown in Fig. 6 suggest that the MOD and HIGH are operating on two different pump function curves with the HIGH operating either at higher contractility and/or preload. The former possibility is consistent with studies in which right ventricular contractility was found to be increased in pulmonary hypertension induced by pulmonary vein banding [66] or embolization [67]. This suggests that the right ventricle is able to compensate for the increased hemodynamic load. The higher outputs at lower pressures produced by PMBV and PPMBV are manifestations of unloading. In both groups NP produced discernible decreases in all the pressures and changed most of the key impedance and wave reflection parameters. Nevertheless, in both groups differences from the CONT persisted. This suggests that the alterations in the vasculature were not all due increased vasoconstriction. With respect to ventricular function, in both groups NP resulted in relatively larger decreases in pressure than increases in cardiac output. These changes are consistent with each group operating on a different function curve compared to the other conditions. Our limited data do not enable us, however, to determine whether the effect of NP is due to lower preload and/or contractility or some combination of the two. Given that the flow is already low at BAS, it is more likely that the NP effect is a reduction in contractility rather than preload. More data are needed to adequately address this issue.

Our results provide evidence for reverse remodeling of the vasculature. For example, in both the MOD and HIGH the decreases in F_0_ from PMBV to PPMBV are  direct evidence. In the SR and Young subgroups, the changes from PMBV to PPMBV in some other parameters are further evidence of reverse remodeling. Additionally, the separate regression results showing that a few parameters had different slopes after PPBMV, compared to BAS or PMBV, is another indication of reverse remodeling. We can only speculate about the positive effects of SR vs AF. It is reasonable to assume, however, that the mechanical and electrophysiological effects of the rhythmic contractions in the left atrium not only help it remodel but these effects are transmitted retrograde to induce reverse remodeling in the distal pulmonary vasculature. Our data do not, however, provide understanding of the mechanism(s) of reverse remodeling.

The apparent ability of the right ventricle to compensate, along with the stronger evidence for remodeling in the HIGH compared to the MOD, suggests that intervening when the MVA becomes moderately to severely narrowed, especially in young patients and those in SR, may be more beneficial than waiting until pulmonary hypertension develops. Nevertheless, even when pulmonary hypertension develops, intervening early may still be beneficial.

#### Pulmonary Hypertension—Other Etiologies

Even though MS is an obstruction beyond the most distal portion of the pulmonary vasculature, it is still instructive to compare our results with previous findings in humans and animals of the effects induced by more proximal obstructions. Similar to our results, in humans with chronic obstructive thromboembolic disease, pulmonary vascular resistance, wave reflections and wavespeed all appeared to be increased above normal but Z_c_ was not [23, 27, 68, 69]. There are many animal studies in which pulmonary vascular obstruction was produced by various means [23, 54, 64, 66–78]. While there is some variability in the findings and different indexes were used, the majority of the results demonstrated changes consistent with our findings. For example there were increases in pulmonary vascular and/or input resistance, wavespeed and reflections and an upward shift in the low frequency portion of the impedance modulus spectrum. Specifically with respect to Z_c_, the majority of the studies reported no change, although two studies reported decreases [72, 77] and three reported increases [67, 70, 74]. The disparate results of the latter three studies could be due to the different methodologies. One study embolized with 3 × 4 mm beads, another used 0.5 mm beads and another used moncrotalline pyrrole. It is not surprising that these produced more proximal effects than embolization with much smaller beads, blood clots or with MS. However, we also cannot rule species and other methodological differences.

### Limitations

There are some limitations to our study that deserve mention. As shown in Table 1, there were significant differences in the mix of sex among the various groups, particularly in the HIGH which had a predominance of females for all conditions. There is evidence not only of a higher prevalence of females with pulmonary hypertension due to various but also that females respond better than males to treatment [79, 80]. Unfortunately, we did not have sufficient numbers in the HIGH to enable analogous subgroup analysis for sex to address this issue. Hence, large scale studies that directly address the effect of sex are needed.

Because many parameters were not normally distributed we used non-parametric statistical tests. Additionally, because of logistical and other reasons, not all of the same patients were in the MOD and HIGH groups at BAS, NP, PMBV and PPMBV. Hence, rather than being able to use the more powerful repeated measures ANOVA types of analyses, we had to consider each group and condition as independent. This is not a big limitation, however, because the Kruskal-Wallis test does not have a repeated measures analog. Rather, this test inherently considers each condition as independent. Therefore, our results, from a statistical standpoint, are on the conservative side.

As described in the Cohort Studies section, the numbers in the PMBV and PPMBV cohorts were smaller than the BAS and NP cohorts. This was partly due to our rather stringent inclusion criteria. In addition there was heterogeneity among the cohorts. Our conclusions would have been stronger had there been more patients and more homogeneity in each cohort. Nevertheless, there are sufficient number of patients to enable our statistical test to yield reasonably robust results. Future studies with larger and more homogeneous cohorts would be helpful to more adequately address these limitations.

Our study population consisted entirely of patients of one race. This is a potential limitation because our results may not be generalizable to a broader population. On the other hand, the racial homogeneity could be viewed as a positive. Otherwise racial heterogeneity could be considered another potential limitation.

Because the CONT were not being examined for cardiac valve problems, we do not have MVA data to enable direct statistical comparisons with the MS groups. Nevertheless, the MVA of both the MOD and HIGH are very similar to the values reported by others with moderate to severe MS in the same pressure range as ours [9, 11, 13, 14, 16–20, 59]. Hence, it is a near certainty that the MVA of both the MOD and HIGH are significantly smaller than the CONT.

We assessed the generalized responsiveness of the pulmonary circulation by acute administration of intravenous NP. First, we do not know whether our findings also pertain after chronic vasodilation. Second, it would be interesting to see if acute or chronic administration of any of other more specific pulmonary vasodilators produces different results than NP. Our goal was, however, simply to assess the role of nonspecific vasodilation rather than attempting to uncover the mechanism(s) of the pulmonary abnormalities, which was beyond the scope of our work. Clearly, more studies are needed to provide detailed insight into the mechanism(s) of action of NP and other vasodilators.

Finally, our data are the first description of the short-term results of pulsatile pulmonary hemodynamics and wave reflections after PMBV. Nevertheless, our follow-up time is still rather short. Several parameters, even though they changed from immediately after PMBV, were still not completely normal at 4 months after PMBV. Longer term follow-up studies are needed to provide more information about whether the short-term remodeling we observed is sustained and/or improved.

## Data Availability

The data that support the findings of this study are not openly available for study participant privacy reasons. They are available from the corresponding author upon reasonable request.
